# Fatty acids from nutrition sources for preterm infants and their effect on plasma fatty acid profiles

**DOI:** 10.1186/s40348-024-00183-9

**Published:** 2024-10-12

**Authors:** Gerhard Fusch, Naomi H. Fink, Niels Rochow, Christoph Fusch

**Affiliations:** 1 Division of Neonatology, Department of Pediatrics, McMaster University, Hamilton, ON Canada; 2 Department of Pediatrics, Paracelsus Medical University (PMU) Nuremberg, Nuremberg General Hospital, Nuremberg, Germany

**Keywords:** Breast milk, Fatty acid, Infant nutrition, Lipid emulsion, Preterm infant

## Abstract

**Background:**

In preterm infants, IV administration of fat is less well tolerated compared to intake via the enteral route, often resulting in hypertriglyceridemia. It is therefore recommended that parenteral fat intake should not exceed 3.5 to 4.0 g/kg/d whereas human milk can provide up to 8 g/kg/d. It is unknown whether such hypertriglyceridemic conditions are caused by a uniform increase of all fatty acids or it is linked to an elevation of distinct fatty acids due to an unbalanced intake. Obviously, both scenarios could potentially influence the formulation of novel lipid solutions for preterm infants. Objective of this exploratory study was to compare fatty acid profiles between a) different nutritional sources and corresponding plasma samples, b) plasma of infants fed breast milk versus those receiving lipid emulsion, and c) plasma of infants with normal versus elevated triglyceride levels.

**Methods:**

Forty-seven preterm infants < 36 weeks of gestation were included; fatty acid profiles were measured in serum samples and corresponding nutritional sources (breast milk and lipid emulsion) using gas chromatography/mass spectrometry.

**Results:**

Compared to breast milk levels, plasma contained significantly lower C8:0, C10:0, C12:0, C14:0, C19:1n9, C18:3n3 (*p* < 0.0001). In contrast, relative abundance of C16:0, C18:0 and C20:4n6 was higher in plasma than in corresponding breast milk samples (*p* < 0.001) and lipid emulsion (*p* < 0.01). Compared to the corresponding lipid emulsion, the abundance of C18:2n6 and C18:3n3 was significantly lower in plasma (*p* < 0.001). Fatty acid profiles in plasma of infants fed breast milk compared to lipid emulsion were not markedly different. Hypertriglyceridemic samples showed elevated levels for C18:1n9 and C16:0 when compared with normotriglyceridemic samples.

**Conclusions:**

Our study reveals that lipid levels in plasma show both depletion and enrichment of distinct fatty acids which do not seem to be closely related to dietary intake. A more detailed understanding of fatty acid flux rates is needed, like the understanding of amino acid metabolism and is supported by the finding that hypertriglyceridemia might be a state of selective fatty acid accumulation. This would allow to develop more balanced diets for intensive care and potentially improve clinical outcomes.

## Background

Preterm birth causes a disruption of nutrient transfer from mother to fetus, rendering the preterm infant dependent on an exogenous source of nutrition that ideally should mimic fetal nutrient accretion rates during the third trimester [1]. For high-risk infants, adequate growth and nutrition in the early postnatal period is essential for long term health. Breast milk (BM) has a balanced fatty acid profile, contains bioactive factors and has long been considered the gold standard for infant nutrition [2, 3]. While breast milk is the ideal nutrition source, it is not always available in the first days of life for preterm infants. Interest in the fatty acid composition of breast milk and that of other products applied in infant nutrition regimens is intensifying [4] because it is not known if, and to what degree, fluxes in dietary fatty acids effect the infant’s metabolism.

Preterm infants receive parenteral lipids in the early postnatal phase alongside the subsequent introduction of enteral feeding volumes. The triglyceride portion of the lipid emulsion is cleared from the blood by lipoprotein lipase, an enzyme that is released from the capillary endothelium [5]. Preterm infants have lower levels of lipoprotein lipase and as a result are at an increased risk for hypertriglyceridemia during periods of parenteral nutrition (PN) [5, 6]. It is unknown whether the absolute amount of lipids administered, elevations in certain fatty acids within the externally administered triglycerides, or levels of lipoprotein lipase alone are responsible for the development of hypertriglyceridemia or if it is a combination of these factors. Maintaining nutritional support that allows for appropriate growth and metabolism while avoiding feeding-related side effects such as hypertriglyceridemia remains a daunting challenge for neonatologists [7].

We have recently reported that infants appear to tolerate higher lipid intake when supplied via the enteral route compared to intravenous administration [8]. Additionally, breast milk fat is more readily absorbed than fat from infant formulas [4]. It is possible that the fatty acid profile of the lipid source itself could play a role in the paradox that a relatively low parenteral lipid intake leads to elevated serum triglyceride levels when compared to enteral lipid administration. Recommended levels and intakes for each single fatty acid have not been clinically established for preterm infants. Furthermore, the limited data comparing dietary fatty acid levels with resulting physiological profiles in infants is not conclusive [4, 9–11]. We therefore compare fatty acid profiles of nutrition sources to the corresponding plasma fatty acid profiles in order to assess three study questions: a) whether preterm infants metabolize fatty acids in proportion to what is supplied in the diet, or in other words if plasma fatty acid levels are correlated with the composition of the nutrition source received, b) whether the plasma fatty acid profiles of infants receiving enteral BM differ from the profiles of infants receiving PN lipid emulsions and c) whether hypertriglyceridemia is characteristically caused or associated with elevations in certain fatty acids.

## Methods

### Study design and sample collection

To test the first two research questions, 47 infants were recruited for this single-centre longitudinal observational study at McMaster University Hospital Level II and Level III neonatal intensive care unit (NICU) between August 2012 and May 2013. Parent(s)/guardian(s) provided written informed consent. Blood samples were obtained on occasion of diagnostic phlebotomies. Time points were requested once during the first 3 days of life, once weekly for one month and once monthly thereafter for the duration of the infant’s stay in the NICU. A corresponding sample of 24-h pooled standard fortified mother’s breast milk (Enfamil, Mead Johnson Nutrition, Glenview, IL) was obtained 48–72 h prior to these blood collections. All fatty acids measured in BM samples collected as part of this study therefore reflect fortified and not native BM.

To test the third research question, a second sample set was studied: hypertriglyceridemic blood samples (triglyceride > 1.7 mmol/L) from preterm infants were identified by the Hamilton Regional Laboratory Medicine Program at McMaster Children’s Hospital and were collected without personal identifiers for analysis [12]. The study was approved by the Hamilton Integrated Research Ethics Board of McMaster University and Hamilton Health Sciences.

### Designation of plasma samples into feeding groups

For testing the first two research questions, study subjects were grouped according to the two nutrition sources administered over a period of 48 h prior to and on the day of blood sampling: a) fortified BM (100%), or b) lipid emulsion (LE) and/or formula (with LE > 50% of total fat intake). The amount of fat (g) intake was calculated using administered feeding volume per day in each infant’s medical record and the following fat contents: fortified BM of 4.4 g/100 mL; formulas: Enfamil preterm 24 kcal of 4.1 g/100 mL; Enfamil preterm 20 kcal of 3.4 g/100 mL; Nutramigen A^+^ of 3.6 g/100 mL; lipid emulsion (Intralipid) of 20 g/100 mL. For the third research question, comparative analyses of normo- versus hypertriglyceridemia, all plasma samples from all nutrition groups were included. Plasma samples were taken 48–72 h after collection of nutritional samples on occasion of the next routine blood collection.

### Sample preparation

For this study, a previously described method was used to extract and esterify fatty acids from BM, LE and plasma [13, 14]. Butylated hydroxytoluene (0.1 mg/mL) was used as a stabilizing agent for the fatty acid methyl esters (FAME).

### Gas chromatography/mass spectrometry

FAME were analyzed on an Agilent 7890 gas chromatographer (Agilent, Santa Clara, CA) with a Supelco IL111 GC column attached to an Agilent 5795 single quad mass detector. A fatty acid standard (Supelco, St. Louis, MO) containing 37 FAME was used to identify retention time and mass/fragmentation pattern of each fatty acid using ChemStation software (Agilent, Santa Clara, CA). The oven temperature profile was set to at 130˚C for 10 min, ramping up 2˚C/minute to 240˚C and held at 240˚C for 2 min at the end of the ramping time. Auxiliary column temperature was maintained at 250 °C. Intra-assay and inter-assay variation was assessed in 10 identical aliquots of BM (coefficient of variation < 1%). Fatty acids were expressed as percent of the total to allow comparisons between different dietary sources and plasma as well as between infant plasma samples.

### Statistical analysis

Analyses were performed using SPSS version 21 (IBM SPSS Statistics, Chicago, Illinois). The analysis results of patient characteristics and outcome variables were summarized using descriptive summary measures: expressed as a mean (standard deviation) for continuous variables and number (percent) for categorical variables. An independent two-tailed t-test was performed to compare means between a) nutrition source (LE or BM) and plasma; b) plasma fatty acid profiles on BM and LE; c) plasma fatty acid profile of normal and hypertriglyceridemic blood samples. Standard deviations were not assumed to be equal and Welch’s correction factor was applied. Resulting compositional differences (difference of means) were calculated with a 95% confidence interval for the difference of the mean.

## Results

**Fig. 1:**
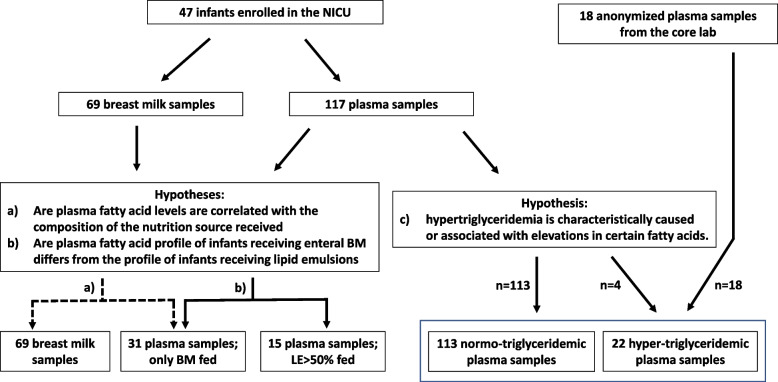
Research question and sample distribution

A total of 47 infants (23 girls, 24 boys) of 36 mothers (7 sets of twins and 2 sets of triplets) with a mean (± SD) gestational age of 30^6/7^ ± 2^4/7^ weeks were included. A total of 117 plasma (113 normo- and 4 hypertriglyceridemic) samples and 69 fortified milk samples were collected. For analysis of hypertriglyceridemic samples, 18 samples were collected additionally (Fig. 1).

Table 1 depicts the fatty acid profile of BM samples received by the preterm infants in the study and of administered lipid emulsion. Levels of caprylic acid (C8:0), capric acid (C10:0), lauric acid (C12:0) and myristic acid (C14:0) were present in BM but not in LE. Compared to LE, BM contained higher proportion of the monounsaturated fatty acid (MUFA) oleic acid (*cis*-C18:1n9) and a lower proportion of linoleic acid (C18:2n6).

**Table 1:** Fatty acid profiles of enteral (69 fortified breast milk samples) and of parenteral (LE) nutritional sources

Fatty acid	Percent of total fatty acids (%)		
	**BM**		**Lipid emulsion**
	**mean**	**min–max**	
**C8:0**	2.3	(0—11.9)	-
**C10:0**	11.1	(0—59.6)	-
**C12:0**	3.9	(0—20.2)	-
**C14:0**	3.3	(0—10.8)	-
**C15:0**	0.1	(0—0.4)	-
**C16:0**	14.7	(0—31.6)	17
**C16:1n7**	1.2	(0—4.6)	-
**C18:0**	5.0	(0—26.0)	6
**cis-C18:1n9**	36.6	(14—62.5)	24
**trans-C18:1n9**	0.1	(0—1.8)	1
**C18:2n6**	18.5	(0—31.6)	45
**C20:0**	0.0	(0—0.5)	-
**C18:3n6**	0.1	(0—0.6)	-
**C20:1n9**	0.0	(0—0.7)	-
**C18:3n3**	1.3	(0—4.7)	5
**C20:2n6**	0.1	(0—0.5)	-
**C20:3n6**	0.3	(0—2.4)	-
**C22:1n9**	0.0	(0—0.9)	-
**C20:4n6**	0.6	(0—7.5)	1
**C22:2n6**	0.9	(0—14.0)	-

Comparison of fatty acid profiles of BM and lipid emulsion

### Comparison of nutritional sources and corresponding plasma fatty acid profiles

Table 2 illustrates the compositional difference between the average plasma fatty acid profile on BM (BM_plasma_) and the fatty acid profile of BM. Mean percentages of fatty acids in BM vs. BM_plasma_ were significantly different at *p* < 0.0001 for C8:0; C10:0; C12:0; C14:0; pentadecanoic acid (C15:0); palmitic acid (C16:0); stearic acid (C18:0); cis-C18:1n9; α-linolenic acid (C18:3n3); and arachidonic acid (C20:4n6). Similar results were obtained from individual analysis of matched pairs of breast milk samples from the mother and corresponding timed plasma fatty acids in the infant (data not shown). The BM contained more medium chain saturated fatty acids (SFA) and the MUFA cis-C18:1n9 while the plasma of infants fed BM contained higher proportions of long chain SFA C16:0 and C18:0 and the polyunsaturated fatty acid (PUFA) C18:3n3 and C20:4n6. BM_plasma_ did not contain any medium chain fatty acids C8:0 and C10:0.

**Table 2:** Compositional differences between saturated (panel A) and unsaturated (panel B) fatty acids in plasma and respective nutritional sources (BM: breast milk, LE: > 50% lipid emulsion, plasma: plasma samples). Fatty acids occurring in proportions less than 0.1% in both groups were not included

A. Saturated fatty acids							
Feeding group	Δ C8:0 [%]	Δ C10:0 [%]	Δ C12:0 [%]	Δ C14:0 [%]		Δ C16:0 [%]	Δ C18:0 [%]
BM vs. BM_plasma_ (n = 31)	2.25 ***	11.06 ***	3.90 ***	3.33 ***		20.56 ***	-19.34 ***
LE vs. LE_plasma_ (n = 15)		-0.02	-0.36	-0.41		-17.99 ***	-11.55 **
BM_plasma_ vs. LE_plasma_		-0.02	-0.36	-0.41		0.33	6.49
B. Unsaturated fatty acids							
Feeding group	Δ cis-C18:1n9 [%]	Δ trans-C18:1n9 [%]	Δ C18:2n6 [%]	Δ C18:3n3 [%]	Δ C20:3n6 [%]	Δ C20:4n6 [%]	Δ C22:2n6 [%]
BM vs. BM_plasma_ (n = 31)	24.95 ***	0.06	-0.19	1.26 ***	-0.02	-7.10 ***	-1.06
LE vs. LE_plasma_ (n = 15)	-0.16	1.03 **	32.95 ***	4.93 ***	-0.25	-8.06 ***	0.04
BM_plasma_ vs. LE_plasma_	-12.35 ***	-0.24	6.40 **	0.18	0.05	-1.12	1.54

^**^p < 0.01; ***p < 0.0001

Table 2 illustrates the comparison of the average plasma fatty acid profile of infants fed > 50% LE (LE_plasma_, *n* = 15) with the LE fatty acid profile of the lipid emulsion administered. The plasma of infants on lipid emulsion contained significantly higher proportions of long chain SFAs C16:0 and C18:0 and the PUFA C20:4n6 compared to the lipid emulsion. The LE itself contained significantly higher proportions of the MUFA elaidic acid (trans-C18:1n9) and the PUFAs C18:2n6 and C18:3n3.

### Comparison of plasma fatty acid profiles between BM and LE feeding groups

Table 2 depicts the compositional differences between average fatty acid levels in plasma of infants that received BM (BM_plasma_, *n* = 31) compared to those that received LE (LE_plasma,_ *n* = 15). BM_plasma_ contained significantly higher proportions of the PUFA C18:2n6 while LE_plasma_ contained a significantly higher proportion of the MUFA cis-C18:1n9. The proportions of other fatty acids were not significantly different in the plasma of the two feeding groups.

### Comparison of fatty acid profiles between normal and hypertriglyceridemic samples

**Fig. 2:**
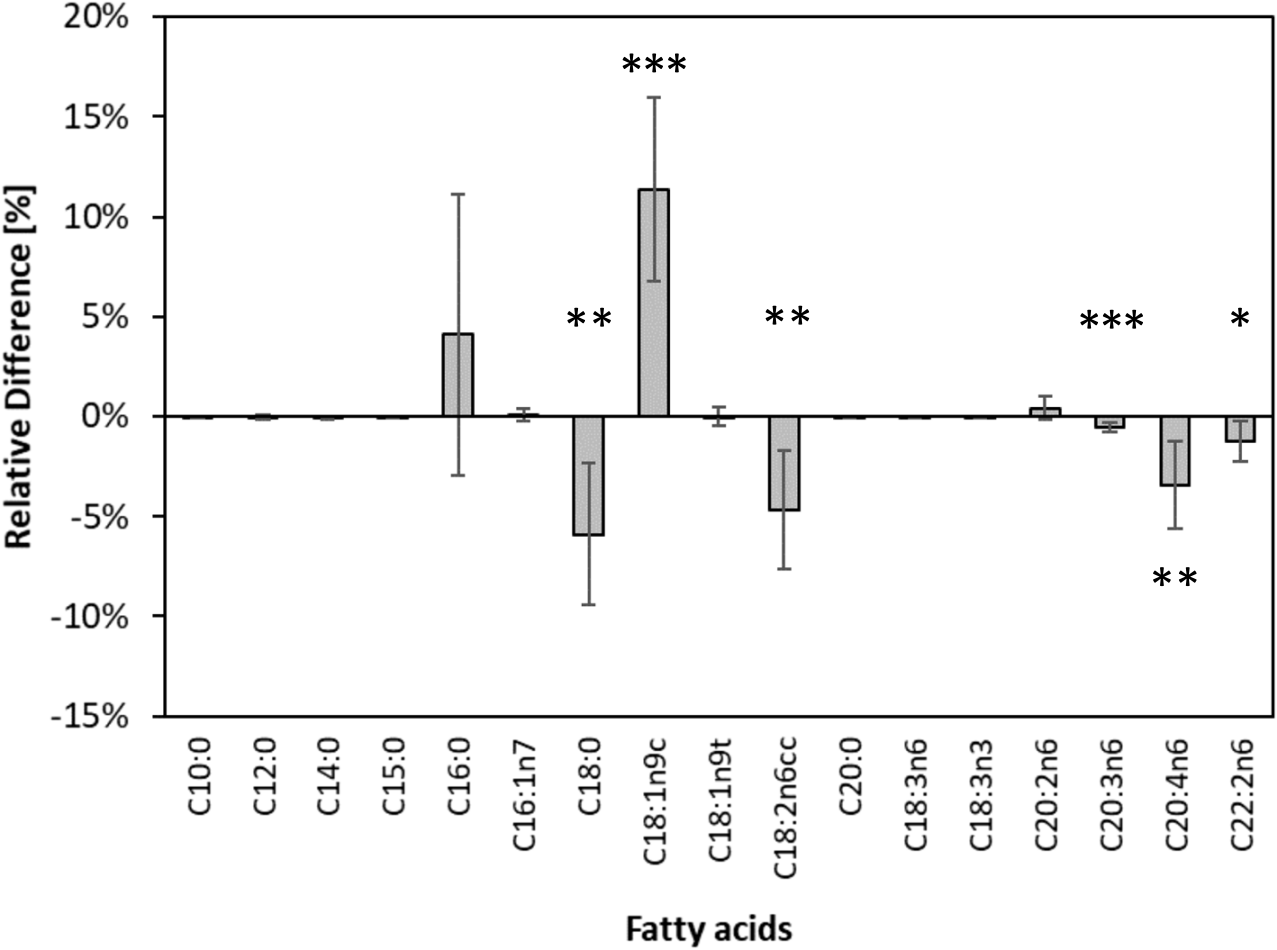
Compositional differences between fatty acids in samples from infants with hyper- (i.e. TG, > 1.7 mmol/L, *n* = 22) and normotriglyceridemic (i.e. TG, ≤ 1.7 mmol/L *n* = 113) samples. Significantly different means are marked with a “***” (*p* < 0.0001), “**” (*p* < 0.01) and “*” (*p* < 0.05)

Figure 2 displays the compositional differences between normal (*n* = 113) and hypertriglyceridemic blood samples (*n* = 22). Mean percentages of fatty acids were significantly different for C18:0 (*p* < 0 0.01); cis-C18:1n9 (*p* < 0.0001); C18:2n6 (*p* < 0.01); C20:3n6 (*p* < 0.0001); C20:4n6 (*p* < 0.01); and C22:2n6 (*p* < 0.05). The samples with normal triglyceride levels had significantly higher proportions of the SFAs C18:0 and the PUFAs C18:2n6; C20:3n6; C20:4n6; and C22:2n6 than the hypertriglyceridemic samples, which only had significantly elevated levels of the MUFA cis-C18:1n9.

## Discussion

Nutrition-to-plasma comparisons presented in this study reveal that individual plasma fatty acid levels are not closely related to levels of the individual nutritional source. Moreover, average plasma fatty acid distribution does not differ between infants receiving either BM or LE. In contrast, fatty acid profiles are significantly different between infants in a normotriglyceridemic compared to a hypertriglyceridemic state. Besides nutritional intervention studies, there was limited research characterizing fatty acid profiles in preterm infants under various physiological conditions. To our knowledge, this is the first study in preterm infants to compare plasma fatty acid profiles in normo- versus hypertriglyceridemia. Moreover, the correlation of actual nutritional samples with the corresponding plasma levels is a major strength of this investigation. Many previous studies have severe methodological drawbacks: some simply use food journals to estimate fatty acid intake and compared this to corresponding plasma LC-PUFA level [15–17]. Others are reporting percent values of fatty acids which do not sum up to 100% [9]. Such data are therefore hard to compare but we feel that the major components of the reported profiles are in a similar order of magnitude compared to the ones we present. To the best of our knowledge there is no study investigating fatty acid profiles of hypertriglyceridemia in this vulnerable population.

Another strength of this analysis is that preterm infants did not receive any exogenous lipids outside of what was supplied by either standard fortified BM or LE. This unique biological setup provides valuable insight in fatty acid metabolism that can hardly be obtained from the mixed diet of older children or adults because it does not allow for such direct nutrition-to-physiology fatty acid comparisons.

Adipose tissue fatty acid composition is considered the gold standard to reflect long-term dietary fatty acid intake patterns, while the fatty acid composition of plasma triglycerides represents dietary fatty acid intake from the previous days [18, 19]. Therefore, we expected that differences in fatty acid profiles between nutrition source and plasma that was obtained 2–3 days later may elucidate which fatty acids are preferentially taken up and which ones remain circulating in the blood in higher levels. Our data reveals that almost all circulating plasma levels in the infants were found to be significantly different from fatty acid profiles provided by different nutritional sources. The same trend existed for individual matched pairs of mother’s milk to resulting infant plasma. Previous studies have reported that medium chain fatty acids (C8:0-C12:0) are readily absorbed and preferentially oxidized, which is consistent with our results given that plasma levels in infants were low to none [20, 21].

A recent study in Canadian preterm infants compared fatty acid levels between nutrition and plasma collected on the same day and found that there were no significant differences other than for linolenic acid (C18:3n3)[9]. In contrast, our results revealed additional significant differences (including C18:3n3) between the overall fatty acid profile of both BM and LE and resulting plasma. Therefore, differences between nutrition and blood levels found in our study could be indicative of a possible physiological preference in preterm infants for the use of some fatty acids such as C8:0-C12:0 as an immediate energy substrate versus a preference for long-term storage.

Consistent elevations of single fatty acids and stark differences between fatty acids in nutrition and circulating plasma levels may also be suggestive of a trend in differential metabolism of certain fatty acid classes (SFA/MUFA/PUFA) or chain length (short/medium/long). While data is lacking for preterm infants, adult data is not conclusive as to whether chain length and degree of saturation of fatty acids influence uptake, oxidation and storage location in the body [22–26]. Furthermore, there is also evidence that elevated amounts of certain fatty acids have the potential to inhibit the metabolism of others [27] and that fat storage depots may preferentially retain certain fatty acids over others [22]. This has the potential to elicit a disruption in equilibrium of fatty acid mobilization and deposition [27] and may partially explain differences between fatty acid profiles in nutrition compared to resulting plasma. The fatty acid composition of individual lipid classes would be an important next step to explore, as there may be differences in proportion between triglycerides and other lipid classes, such as phospholipids and cholesterol esters. This was not examined as a part of this exploratory pilot project due to limited sample volume, but this data would expand the knowledge of uptake, oxidation, and storage patterns of fatty acids from the diet to inform what the ideal fatty acid profile should be in breast milk substitutes for preterm infants.

While the fatty acid profiles of BM and lipid emulsion are markedly different, the plasma fatty acid profile of infants receiving BM compared to the plasma in those receiving primarily LE was relatively similar. This suggests that plasma fatty acid profiles in preterm infants exhibit some degree of stability and may not directly reflect the fatty acid profile of the nutrition source supplied. This requires further research in preterm infants, as a recent study in healthy term infants observed significant differences in red blood cell SFA, MUFA and some PUFA between infants receiving BM compared to two experimental infant formulas [4].

Free fatty acids in plasma provide a more accurate measure of lipid tolerance but due to the complexity of the assay, are not routinely measured and triglyceride levels are assessed instead [28]. If preterm infants do metabolize and clear certain fatty acids in dietary sources differently, absolute levels of individual fatty acids or ratios between classes (i.e. SFA/MUFA/PUFA or short/medium/long chain) may be a better monitor of safety than just using triglyceride levels. Our data indicates that differences exist between fatty acid profiles in plasma with normal compared to high triglyceride levels, but causative information cannot be drawn due to the exploratory nature of this study. A larger sample size ideally from a multi-centre study comparing absolute fatty acid levels from preterm infants under several lipid emulsions would be required to investigate if elevations in single fatty acids are associated with or predictive of hypertriglyceridemia. If such trends are found, consideration can be given to the amount and type of each fatty acid in infant formulas, human milk fortifier and LE preparations.

Our study reveals a discordance between nutrition and circulating fatty acid levels in preterm infants. However, the differences between plasma fatty acid profiles of infants receiving different nutritional sources with different fatty acid profiles, BM and LE, are not striking while the difference between normal and hypertriglyceridemic sample is. This suggests that we need to understand better flux rates of individual fatty acids, a research area comparable to what has been done in amino acid metabolism. This might also help to understand why the metabolic tolerance of parenterally administered fatty acids is significantly lower compared to application via the enteral route. For example, differences in bioavailability or preferred retention of fatty acids in certain tissue lipids may play a role in the observed lack of correlation between nutrition and blood samples in this study.

Currently, many high impact studies report fatty acids levels using relative abundance [29, 30]. This approach however is somewhat confusing as the relative change in one fatty acid will influence the relative abundance of the rest. Absolute quantification of fatty acid levels in nutritional sources and plasma is more robust and reflects the true changes and is therefore more appropriate to study metabolism and flux of fatty acids. However, there are only a few methods being published and the approach is much more sophisticated compared to assessment of relative abundances. We currently are aiming to establish a procedure for absolute quantification of fatty acids in biological samples to examine this relationship [31].

In summary, the results of this pilot study support continued research in this area. A multi-centre study would allow for an examination of the effect of other nutritional products such infant formula and next-generation lipid emulsions on plasma fatty acid levels and could reveal if fatty acid metabolism varies according to the composition of the dietary source.

## Conclusion

Fatty acid profiles of different nutritional sources such as formula, lipid emulsions or breast milk are highly variable. The fatty acid profiles in blood of the preterm infants studied do not correlate with fatty acid profiles of their nutritional sources. Hypertriglyceridemia is in part caused by accumulation of distinct fatty acids. More research is needed to better understand fatty acid flux rates and metabolism.

### Addendum

During the review process, a number of questions and comments came up dealing with lipid nutrition that shall be addressed in the following paragraph.

It has been asked whether there is an impact of inter- und intraindividual variations of total breast milk fat content on fatty acids compositions and whether it changes during the lactation over time. Such changes are well known for protein content of human milk and there is a plethora of published studies on this subject. However, as much as we feel this question to be of interest, it can be easily understood that—in light of the data matrix being needed – this is definitively a study on its own to be conducted in the near future.

On another note, it was being criticized that the populations of normal- and hyper-triglyceridemic infants have not been described more in demographic details and that lipid intakes of hyper-triglyceridemic infants were not given. In this context, we would like to elucidate that it was not the purpose of the study to assess demographic factors that lead to lipid intolerance, nor which patients will be at risk. This would have required a completely different study design. The mere purpose of this study was to look at fatty acid distribution patterns in normo- and hypertriglyceridemic samples to assess whether hypertriglyceridemia is caused or associated with an accumulation of some fatty acids.

It was also questioned by one reviewer whether fatty acid fluxes should be of interest at all. We firmly believe that obtaining a better understanding of fatty acid fluxes is key to improve the metabolic safety. The major question—unsolved so far – is whether hypertriglyceridemia is caused by a uniform increase of all fatty acids or by a disproportionate increase of only a few fatty acids. Such data are not available for this population. If hypertriglyceridemia would have been caused by the latter mechanism, this would have an impact on the composition of the fatty acid profile provided. In this respect also flux studies would become relevant—in analogy to metabolic studies on amino acid fluxes to avoid toxic accumulation which have greatly improved our understanding.

## Data Availability

The datasets generated and analyzed during the current study are available from the corresponding author on reasonable request.

## Abbreviations

BM: Breast milk
FAME: Fatty acid methyl ester
LE: Lipid emulsion
MUFA: Monounsaturated fatty acid
NICU: Neonatal intensive care unit
PN: Parenteral nutrition
PUFA: Polyunsaturated fatty acid
SFA: Saturated fatty acid

## Data availability

No datasets were generated or analysed during the current study.

## References

[1] Hanebutt FL, Demmelmair H, Schiessl B, Larque E, Koletzko B (2008) Long-chain polyunsaturated fatty acid (LC-PUFA) transfer across the placenta. Clin Nutr 27(5):685–693 18639956 10.1016/j.clnu.2008.05.010

[2] Berenhauser AC, Pinheiro do Prado AC, da Silva RC, Gioielli LA, Block JM (2012) Fatty acid composition in preterm and term breast milk. Int J Food Sci Nutr 63(3):318–325 22023571 10.3109/09637486.2011.627843

[3] Lonnerdal B (2016) Bioactive proteins in human milk: health, nutrition, and implications for infant formulas. J Pediatr 173(Suppl):S4–9 27234410 10.1016/j.jpeds.2016.02.070

[4] Visentin S, Vicentin D, Magrini G, Santandreu F, Disalvo L, Sala M et al (2016) Red blood cell membrane fatty acid composition in infants fed formulas with different lipid profiles. Early Hum Dev 100:11–15 27391868 10.1016/j.earlhumdev.2016.05.018

[5] Valentine CJ, Puthoff TD (2007) Enhancing parenteral nutrition therapy for the neonate. Nutr Clin Pract 22(2):183–193 17374792 10.1177/0115426507022002183

[6] Lim MS, Choi CW, Kim BI, Yang HR (2013) Clinical factors affecting lipid metabolism and optimal dose of heparin in preterm infants on parenteral nutrition. Pediatr Gastroenterol Hepatol Nutr 16(2):116–122 24010115 10.5223/pghn.2013.16.2.116 PMC3760703

[7] Puntis JW (2006) Nutritional support in the premature newborn. Postgrad Med J 82(965):192–198 16517801 10.1136/pgmj.2005.038109 PMC2563699

[8] Rochow N, Moller S, Fusch G, Drogies T, Fusch C (2010) Levels of lipids in preterm infants fed breast milk. Clin Nutr 29(1):94–99 19666201 10.1016/j.clnu.2009.07.002

[9] Hossain Z, MacKay D, Friel JK (2016) Fatty acid composition in feeds and plasma of Canadian premature infants. J Pediatr Gastroenterol Nutr 63(1):98–102 26835902 10.1097/MPG.0000000000001134

[10] Wang Y, Feng Y, Lu LN, Wang WP, He ZJ, Xie LJ et al (2015) The effects of different lipid emulsions on the lipid profile, fatty acid composition, and antioxidant capacity of preterm infants: a double-blind, randomized clinical trial. Clin Nutr 35(5):1023–1031 26561301 10.1016/j.clnu.2015.10.011

[11] Amusquivar E, Sanchez M, Hyde MJ, Laws J, Clarke L, Herrera E (2008) Influence of fatty acid profile of total parenteral nutrition emulsions on the fatty acid composition of different tissues of piglets. Lipids 43(8):713–722 18491157 10.1007/s11745-008-3180-7

[12] Expert Panel on Detection Evaluation and Treatment of High Blood Cholesterol in Adults (2001) Executive summary of the third report of the National Cholesterol Education Program (NCEP) expert panel on detection, evaluation, and treatment of high blood cholesterol in adults (Adult treatment panel III). JAMA 285:2486–2497 11368702 10.1001/jama.285.19.2486

[13] Fidler N, Sauerwald T, Pohl A, Demmelmair H, Koletzko B (2000) Docosahexaenoic acid transfer into human milk after dietary supplementation: a randomized clinical trial. J Lipid Res 41(9):1376–1383 10974044

[14] Fidler N, Sauerwald TU, Koletzko B, Demmelmair H (1998) Effects of human milk pasteurization and sterilization on available fat content and fatty acid composition. J Pediatr Gastroenterol Nutr 27(3):317–322 9740204 10.1097/00005176-199809000-00009

[15] Uusitalo L, Nevalainen J, Salminen I, Ovaskainen ML, Kronberg-Kippila C, Ahonen S et al (2013) Fatty acids in serum and diet–a canonical correlation analysis among toddlers. Matern Child Nutr 9(3):381–395 22066932 10.1111/j.1740-8709.2011.00374.x PMC6860655

[16] Nikkari T, Luukkainen P, Pietinen P, Puska P (1995) Fatty acid composition of serum lipid fractions in relation to gender and quality of dietary fat. Ann Med 27(4):491–498 8519511 10.3109/07853899709002458

[17] Nilsson AK, Löfqvist C, Najm S, Hellgren G, Sävman K, Andersson MX et al (2019) Influence of human milk and parenteral lipid emulsions on serum fatty acid profiles in extremely preterm infants. JPEN 43(1):152–161. 10.1002/jpen.1172 10.1002/jpen.1172 PMC6437763 29679529

[18] Hodson L, Skeaff CM, Fielding BA (2008) Fatty acid composition of adipose tissue and blood in humans and its use as a biomarker of dietary intake. Prog Lipid Res 47(5):348–380 18435934 10.1016/j.plipres.2008.03.003

[19] Wolk A, Furuheim M, Vessby B (2001) Fatty acid composition of adipose tissue and serum lipids are valid biological markers of dairy fat intake in men. J Nutr 131(3):828–833 11238766 10.1093/jn/131.3.828

[20] Takeuchi H, Sekine S, Kojima K, Aoyama T (2008) The application of medium-chain fatty acids: edible oil with a suppressing effect on body fat accumulation. Asia Pac J Clin Nutr 17(Suppl 1):320–323 18296368

[21] Papamandjaris AA, MacDougall DE, Jones PJ (1998) Medium chain fatty acid metabolism and energy expenditure: obesity treatment implications. Life Sci 62:1203–1215 9570335 10.1016/s0024-3205(97)01143-0

[22] Cantwell MM (2000) Assessment of individual fatty acid intake. Proc Nutr Soc 59(2):187–191 10946786 10.1017/s0029665100000203

[23] Peters JC, Holcombe BN, Fuller LK, Webb DR (1991) Caprenin 3. Absorption and caloric value in adult humans. J Am CollToxicol 10:357–367

[24] Lien EL (1994) The role of fatty acid composition and positional distribution in fat absorption in infants. J Pediatr 125(5 Pt 2):S62–S68 7965455 10.1016/s0022-3476(06)80738-9

[25] Garaulet M, Hernandez-Morante JJ, Lujan J, Tebar FJ, Zamora S (2006) Relationship between fat cell size and number and fatty acid composition in adipose tissue from different fat depots in overweight/obese humans. Int J Obes (Lond) 30(6):899–905 16446749 10.1038/sj.ijo.0803219

[26] Ramirez M, Amate L, Gil A (2001) Absorption and distribution of dietary fatty acids from different sources. Early Hum Dev 65(Suppl):S95–S101 11755040 10.1016/s0378-3782(01)00211-0

[27] Farquharson J, Cockburn F, Patrick WA, Jamieson EC, Logan RW (1993) Effect of diet on infant subcutaneous tissue triglyceride fatty acids. Arch Dis Child 69(5):589–593 8257182 10.1136/adc.69.5.589 PMC1029625

[28] Duggan C, Watkins JB, Walker WA (2008) Nutrition in pediatrics: basic science and clinical applications, 3rd edn. BC Decker Inc., Hamilton, Ontario, Canada

[29] Smithers LG, Gibson RA, McPhee A, Makrides M (2008) Effect of two doses of docosahexaenoic acid (DHA) in the diet of preterm infants on infant fatty acid status: results from the DINO trial. Prostaglandins Leukot Essent Fatty Acid 79(3–5):141–146 10.1016/j.plefa.2008.09.015 18951004

[30] Pupillo D, Simonato M, Cogo PE, Lapillonne A, Carnielli VP (2016) Short-term stability of whole blood polyunsaturated fatty acid content on filter paper during storage at -28 degrees C. Lipids 51(2):193–198 26749585 10.1007/s11745-015-4111-z

[31] Choi A, Fusch G, Rochow N, Abed H, Fusch C (2016) Profiling fatty acid concentrations in hypertriglyceridemic plasma from preterm infants. Monatsschr Kinderheilkd 164(Supp 3):230–390

